# Impact of vaccine delays at the 2, 4, 6 and 12 month visits on incomplete vaccination status by 24 months of age in Quebec, Canada

**DOI:** 10.1186/s12889-018-6235-6

**Published:** 2018-12-11

**Authors:** Marilou Kiely, Nicole Boulianne, Denis Talbot, Manale Ouakki, Maryse Guay, Monique Landry, Chantal Sauvageau, Gaston De Serres

**Affiliations:** 1 Institut national de santé publique du Quebec, 2400 d’Estimauville Avenue, Quebec, G1E 7G9 Canada; 2 Department of Social and Preventive Medicine, Laval University, Quebec, Canada; 3 CHU de Québec-Université Laval, Quebec, Canada; 4 Department of Community Health Sciences, Sherbrooke University, Quebec, Canada; 5 Centre intégré de Santé et Services Sociaux de la Montérégie-Centre, Quebec, Canada; 6 Ministère de la Santé et des Services Sociaux, Quebec, Canada

**Keywords:** Delay, Timeliness, Vaccine coverage, Vaccination, Infant

## Abstract

**Background:**

Timeliness in the administration of recommended vaccines is often evaluated using vaccine delays and provides more information regarding the susceptibility of children to vaccine-preventable diseases compared with vaccine coverage at a given age. The importance of on-time administration of vaccines scheduled at the first visit is well documented, but data are scarce about the impact of vaccine delays at other visits on vaccination status by 24 months of age. Using vaccine delays for the first three doses of DTaP-containing vaccines and for the first dose of measles-containing vaccines as markers of timeliness at the 2, 4, 6 and 12 month visits, we estimated the proportion of incomplete vaccination status by 24 months of age attributable to a vaccine delay at each of these visits.

**Methods:**

We used the data from six cross-sectional coverage surveys conducted in the Province of Quebec from 2006 to 2016 which included 7183 children randomly selected from the universal health insurance database. A vaccine dose was considered delayed if received 30 days or more after the recommended age. The impact of new vaccine delays at each visit on incomplete vaccination status by 24 months of age was estimated with the attributable risk in the population.

**Results:**

The proportion of children with vaccine delay was 5.4% at 2 months, 13.3% at 4 months, 23.1% at 6 months and 23.6% at 12 months. Overall, 72.5% of all 2-year-old children with an incomplete status by 24 months were attributable with a vaccine delay, of which 16.1% were attributable with a first vaccine delay at 2 months, 10.6% at 4 months, 14.0% at 6 months and 31.8% at 12 months.

**Conclusions:**

While great emphasis has been put on vaccine delays at the first vaccination visit, the prevalence of vaccine delays was greater with later visits and most children with an incomplete vaccination status by 24 months had a vaccine delay occurring during these later visits. Interventions to improve timeliness should address vaccine delays at each visit and not only focus on the first visit.

**Electronic supplementary material:**

The online version of this article (10.1186/s12889-018-6235-6) contains supplementary material, which is available to authorized users.

## Background

Vaccine coverage is a common indicator used for the evaluation and monitoring of vaccination programs. It evaluates the proportion of individuals who have received all the recommended vaccines, regardless of the timeliness [1]. Timely vaccination is increasingly used to monitor vaccination programs. By assessing the interval between the recommended age and the age at which a dose was administered, this indicator provides a better estimate of the period during which children are protected [2–4]. Timely vaccination is often evaluated using vaccine delays for which several definitions exist. The definition most commonly used is a delay of 30 days or more after the recommended age for each dose [3–10]. A vaccine delay for a dose may impact on-time administration of subsequent doses and increase the child’s risk of disease targeted by the vaccine [11, 12].

In Quebec (Canada), vaccines recommended to children are free of charge and are mostly administered by public health clinics. In 2005, a survey conducted by the Quebec Ministry of Health reported that vaccine delays to the childhood schedule were occurring in public health clinics of several regions of the province. These delays were attributed to a variety of factors including the introduction of new vaccine programs in the early 2000’s [13, 14], missed opportunities due to providers reluctance to administer several injections at the same visit and barriers like difficulty to get a vaccination appointment. In response, monitoring of vaccine delays has been introduced in 2006 in public health clinics for the 2-month and the 12-month visits. To improve the 2-month visit timeliness, appointment periods with priority given to first visits were added and activities such as reminders and recalls were implemented. For children with vaccine delays, an accelerated vaccination schedule can be applied using the minimum intervals between the vaccine doses instead of the intervals recommended for routine vaccination.

Since 2006, vaccine coverage surveys have been conducted every 2 years in children aged 1 and 2 years. The 2006 survey found that only 17% of 24-month old children had received all recommended vaccine doses with no delays. This survey as well as other studies had shown that vaccine delays of 30 days or more for vaccines scheduled at the first visit at 2 months of age were associated with vaccine delays at later visits or with an incomplete vaccination status by 24 months of age [9, 15–18]. However there are scarce data regarding the frequency of vaccine delays at other visits and their impact on vaccine coverage by 24 months of age. Using Quebec vaccine coverage surveys from 2006 to 2016, we estimated the proportion of children with vaccine delays at 2, 4, 6 and 12 months and the proportion of incomplete vaccination status by 24 months of age attributable to a vaccine delay at each of these visits. To identify more vulnerable populations, factors associated with vaccine delays at each vaccination visit from 2 to 12 months were assessed.

## Methods

### Study population and survey design

The present study is based on the data from six cross-sectional surveys conducted in Quebec in 2006, 2008, 2010, 2012, 2014 and 2016 [15, 19–23]. With the authorization of the Quebec Access to Information Commission, children in these studies were randomly selected from the Quebec Universal Health Insurance database which includes all children from the province. Each survey included a “1-year cohort” and a “2-year cohort” with children aged 15 to 17 months and 24 to 26 months respectively at the time of the survey. With surveys conducted every other year and cohorts defined by year of birth (e.g. in the survey conducted at the beginning of 2006, the 2-year cohort included children born in 2003 and those in the 1-year cohort were born in 2004), the six surveys assessed the immunization data in 12 different birth cohorts (born between 2003 and 2014). The surveys invited approximately 1000 children in each cohort (only 600 for 2006), a number deemed sufficient to obtain a precision of ±3% in the vaccine coverage for each survey assuming a response rate of about 60% [24]. Were excluded children living in the two northern regions of the province.

Questionnaires were sent by mail and were filled out by parents or legal guardians. A postal reminder was sent to non-respondents 2 weeks and 4 weeks later. In the absence of response, parents were called directly 2 weeks after the last postal reminder and those with unknown phone number or not reached by phone received another questionnaire by mail. Respondents were invited to transcribe on the questionnaire the information available in their child’s vaccination booklet (i.e. vaccine names, date of vaccination and vaccine providers). The questionnaire also collected information on the characteristics of the child, the mother and the parents. For children without vaccination booklets and those with information incomplete or inconsistent with the provincial vaccine schedule, vaccine providers were contacted to collect/validate the information. Only written information on doses from parents or vaccine providers were accepted. Each survey was approved by the Ethic Board Committee of the CHU de Quebec-Université Laval Hospital and written consent was obtained from all the participants.

### Vaccination schedule

Since 2004, many new vaccines had been introduced in the Quebec’s vaccination schedule (Table 1).The pneumococcal conjugate vaccine (PCV) was introduced in December 2004 with 3 doses scheduled at 2, 4 and 12 months. A single dose of monovalent varicella vaccine at 12 months of age was introduced in 2006 and replaced in 2008 by the combined measles, mumps, rubella and varicella vaccine (MMRV). Since November 2011, children are receiving two doses of rotavirus vaccine at 2 and 4 months of age. Finally, in June 2013, hepatitis B vaccine program was launched and the combined vaccine against diphtheria, acellular pertussis, tetanus, polio, *Haemophilus influenzae* type b and Hepatitis B (DTaP-IPV-Hib-HB) replaced the pentavalent vaccine DTaP-IPV-Hib in the schedule at 2, 4 and 18 months of age.

**Table 1:** The Quebec vaccine schedule before 24 months of age and definition of vaccine delays used in the analysis

Recommended age	Vaccine schedule by date of several changes made					Minimum acceptable age	Age of child when vaccine delays were initiated
	January 2006	May 2008	November 2011	May 2013	June 2013		
2 months	DTaP-IPV-Hib, PCV	DTaP-IPV-Hib, PCV	DTaP-IPV-Hib, PCV **Rota**	DTaP-IPV-Hib, PCV Rota	**DTaP-IPV-Hib-HB**, PCV, Rota	42 days	≥ 91 days (61 days + 30.43 days)
4 months	DTaP-IPV-Hib, PCV	DTaP-IPV-Hib, PCV	DTaP-IPV-Hib, PCV **Rota**	DTaP-IPV-Hib, PCV Rota	**DTaP-IPV-Hib-HB**, PCV, Rota	Previous doses + 28 days	≥ 152 days (122 days + 30.43 days)
6 months	DTaP-IPV-Hib	DTaP-IPV-Hib	DTaP-IPV-Hib	DTaP-IPV-Hib	DTaP-IPV-Hib	Previous dose + 28 days	≥ 213 days (183 days + 30.43 days)
12 months	MMR + varicella monovalent vaccine, PCV, Men-C-C	**MMRV** PCV, Men-C-C	MMRV PCV, Men-C-C	**MMR** PCV, Men-C-C	MMR PCV, Men-C-C	365 days	≥ 395 days (365 days + 30.43 days)
18 months	DTaP-IPV-Hib, MMR	DTaP-IPV-Hib, MMR	DTaP-IPV-Hib, MMR	DTaP-IPV-Hib, **MMRV**	**DTaP-IPV-Hib-HB**, MMRV	DTaP-IPV-Hib ± HB: Previous dose + 183 days MMR ± V: Previous dose+ 28 days	≥ 578 days (548 days + 30.43 days)

Source: Ministère de la Santé et des Services sociaux. Protocole d’immunisation du Québec, 6^e^ edition: http://publications.msss.gouv.qc.ca/msss/document-000105

Changes in the vaccines used appear in boldface

### Outcomes

Birth and vaccination dates were used to compute age at vaccination in days. A dose was administered on-time if received within 30 days of the recommended age and was considered delayed after this period (Table 1). Vaccine delays were assessed for each of the first three doses of the DTaP-containing vaccine (DTaP) recommended at 2, 4 and 6 months of age and for the first dose of the measles-containing vaccine (Measles) recommended at 12 months. As most recommended vaccines are administered at the same visit, these vaccines have been used as markers of all vaccines administered at each visit. A vaccine delay at one dose is frequently followed by a vaccine delay at the following dose(s). A new vaccine delay was defined as a delay occurring among children whose previous dose(s) were on-time (e.g. Delay at 4 months among children with no delay at 2 months).

Given the changes in the Quebec vaccination schedule over the study period, the vaccination status was defined for antigens common to all surveys (DTaP-IPV-Hib, Men C-C and measles-mumps-rubella). A complete vaccination status by 24 months of age was defined as having received 4 doses of DTaP-IPV-Hib, 1 dose of Men-C-C vaccine and 2 doses of MMR vaccine before 2 years of age. Otherwise, the vaccination status was incomplete.

### Statistical analysis

All analyses were performed with Statistical Analysis System (SAS Institute Inc. Carry, NC, version 9.4). Proportions were compared using Chi-square test or Chi-square test for trend when appropriate. Missed opportunities at 2 and 12-month visits occurred when a vaccine-eligible child did not receive all recommended vaccines at the same vaccination date [25]. Other independent variables included data regarding the child, the family and the vaccine provider. The data on vaccine provider at the 2 month visit were used for the analysis of vaccine delays at 2, 4 and 6 months and the data on vaccine provider for all vaccination visits from 2 to 12 months were used for the analysis of vaccine delays at 12 months.

As vaccine delays occurred before 15 months of age, the analyses on factors associated with new vaccine delays included all participants to maximize the statistical power. In contrast, as children in the “1-year cohort” had not yet reached 2 years of age, the impact of new vaccine delays at 2, 4, 6 or 12 months on vaccination status by 24 months of age was estimated only with children in the “2-year cohort”.

The impact of new vaccine delays at 2, 4, 6 and 12 month on vaccination status by 24 months of age was estimated with a robust Poisson multivariable regression. This alternative to logistic regression for binary outcomes directly estimates relative risk without risking convergence issues associated with log-binomial regressions [26–28]. The SAS GENMOD procedure was used with a log link and a robust estimator of the standard errors. All potential factors associated to incomplete vaccine coverage and to vaccine delay were included in the model without any selection procedure [29]. Risk-ratio modification by the survey year was assessed with an interaction term between vaccine delay and survey year in the model. A model based standardization approach was used to estimate adjusted risk difference (RD) in incomplete vaccination status by 24 months of age between children with and without vaccine delays [30]. Briefly, using predicted probabilities of the model, the standardized risk of outcome considering all children exposed (with vaccine delays) was estimated. The standardized risk considering all children unexposed (without vaccine delays) was also estimated similarly. The standardized RD was obtained as the difference between these two quantities. Adjusted attributable risks in the population (ARp) were estimated for delays at each dose also using a model based standardization approach. That is, ARp were estimated as one minus the ratio of the predicted risk of outcome considering all children unexposed over the observed risk of outcome [31]. Confidence intervals for RD and ARp were obtained through the percentile method by performing non-parametric bootstrap with 1000 samples [32, 33].

Factors associated with new vaccine delays were assessed for each dose with the same SAS procedure used to estimate the effect of vaccine delays on vaccination status by 24 months of age. A backward procedure with a *p* value < 0.05 was used for the selection criteria. The year of survey was kept in multivariable models to ensure face validity.

## Results

### Characteristics of participants, vaccine delays and vaccine coverage

Of the 11,200 children invited to participate since 2006, the participation rate was 70% (844/1200) in 2006, 64% (1282/2000) in 2008, 62% (1233/2000) in 2010, 73% (1459/2000) in 2012, 69% (1384/2000) in 2014 and 65% (1295/2000) in 2016. Children born outside Quebec were excluded from the analysis because they were exposed to a different vaccine schedule (*n* = 140 for the 1-year cohorts and *n* = 174 for the 2-year cohorts). This left a total of 3675 children in the 1-year cohorts and 3508 children in the 2-year cohorts (95% of participants for both cohorts) for the analysis.

Forty-four percent of participant children were the first child in the family, 76.9% attending daycare and the majority were vaccinated in public health clinics (66.7%). Most parents lived with a partner (91.9%) and most mothers have completed a college or university degree (70.5%). Missed opportunities occurred in 3.1% of children at the 2-month visit for which two injections were to be administered and in 14.8% at the 12-month visit where three injections were recommended (from 31.1% in 2006 to 7.0% in 2016). All characteristics of children participants are presented in Additional file 1.

**Fig. 1:**
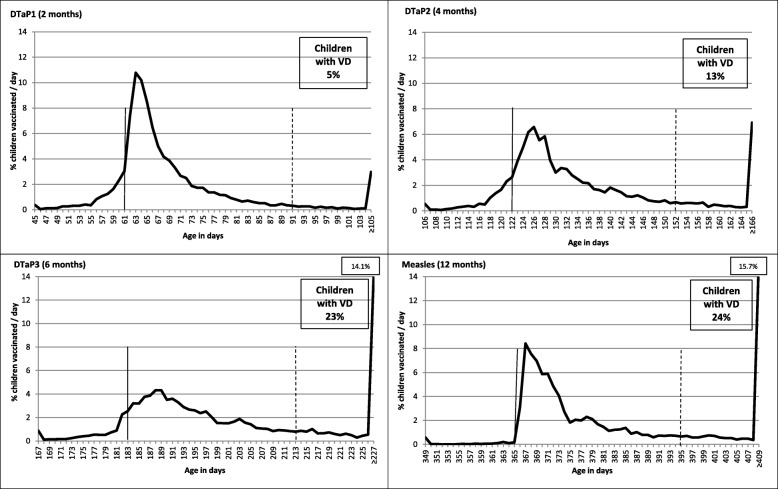
Proportion of children vaccinated according to age in days at 2–4-6 and 12 months, 2006–2016*. *Both cohorts included. The vertical reference lines indicate recommended age at vaccination for each visit and dotted lines indicate age when a dose becomes delayed

**Fig. 2:**
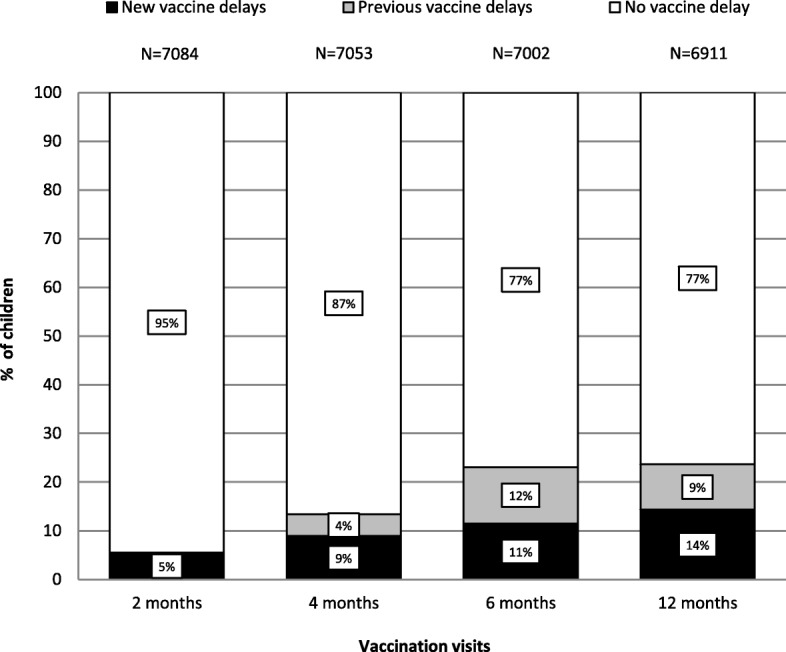
Proportion of children with and without vaccine delays at 2–4-6 and 12 month visits, 2006–2016*. *Both cohorts included. DTaP1 is recommended at 2 months, DTaP2 at 4 months, DTaP3 at 6 months and the first measles-containing vaccine at 12 months

**Fig. 3:**
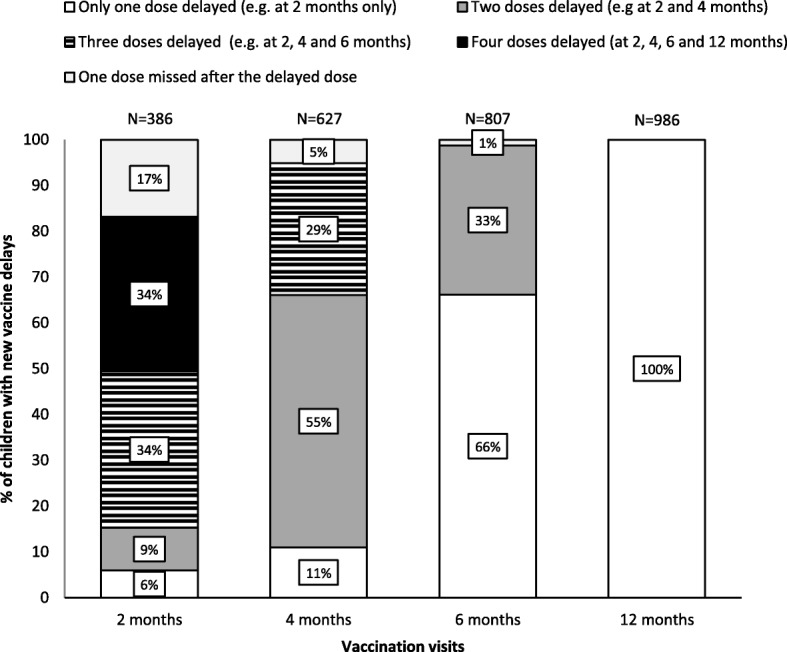
Distribution of total vaccine delays by visit with first (new) vaccine delay, 2006–2016*. *Both cohorts included. DTaP1 was recommended at 2 months, DTaP2 at 4 months, DTaP3 at 6 months and the first measles-containing vaccine at 12 months**.** Categories of vaccine delays were mutually exclusive. The maximum number of delayed doses was 4 for a first (new) delay at 2 months, 3 at 4 months, 2 at 6 months and 1 at 12 months

The proportion of children vaccinated by age in days for each dose is presented in Fig. 1. At 2 months, a greater proportion of children received their DTaP1 close to the recommended age compared to subsequent visits. At 6 months, 14.1% of children received DTaP3 after 227 days. The proportion of new vaccine delays increased from 5.4% at 2 months to 14.3% at 12 months (Fig. 2). Among children who experienced vaccine delays at 2 months (*n* = 386), 77.2% had vaccine delays at other visits (298/386) (Fig. 3) and 33.7% had vaccine delays at each of the next three visits. At 4 months there were 2.4 times more vaccine delays (*n* = 941) than at 2 months of which 66.6% (627) were new vaccine delays. Among these new vaccine delays, 84.1% experienced vaccine delays at subsequent visits (527/627). At 6 months, there were 4.2 times (1617) more vaccine delays than at the 2 month visit and 49.9% (807) were new vaccine delays. At 12 months, the number of vaccine delays was similar to that at 6 months (1624) and 60.7% (986) were new vaccine delays (Fig. 2 and Table 2). In children with a vaccine delay for DTaP doses, instead of using the minimum interval recommended for accelerated schedule to prevent additional vaccine delays, the majority received their next dose with 2-month intervals as recommended for routine schedule (see Additional file 2).

The characteristics of participants according to vaccine delays at 2, 4, 6 and 12 months of age are presented in Table 2. The proportion of children with a vaccine delay increased with age, from 5.4% at the 2-month visit to 23.1% at the 6-month visit and 23.6% at the 12-month visit. Among children with a vaccine delay, the interval between 30 days after the recommended age and the time they received their dose was a median of 17 days for DTaP1 at 2 months, 10 days for DTaP2 at 4 months, 13 days for DTaP3 at 6 months and 21 days for measles at 12 months. Overall, 39% of children experienced at least one vaccine delay at one of the four visits, decreasing from 50% in 2006 to 30% in 2016. In univariate analysis, all characteristics except the sex were associated with new vaccine delays for at least one of the visits (Table 2). Between 2006 and 2016, for children immunized in public health clinics, the proportion with new vaccine delays decreased from 9.1 to 3.6% at the first visit, from 13.7 to 6.5% at the 4 month visit, from 20.3 to 11.0% at the 6 month visit and from 31.0 to 11.2% at the 12 month visit. In contrast, no significant differences were observed for children vaccinated in medical clinic/hospital for the 4 and 6 month visits (see Additional file 3).

**Table 2:** Characteristics of the participants according to new vaccine delays (2–4-6 and 12 months), 2006–2016 (*N* = 7183)^a^

	Vaccine delays											
	DTaP1 (2 month visit)			DTaP2 (4 month visit)			DTaP3 (6 month visit)			Measles (12 month visit)		
	Frequency^b^	%	*P* value^c^	Frequency^b^	%	*P* value^c^	Frequency^b^	%	*P* value^c^	Frequency^b^	%	*P* value^c^
Dose not administered	99			130			181			272		
Total vaccine delays	386/7084^d^	5.4%		941/7053^d^	13.3%		1617/7002^d^	23.1%		1634/6911^d^	23.6%	
New vaccine delays	386/386	100%		627/941	66.7%		807/1617	49.9%		986/1634	60.3%	
Denominator used for the analysis	*N* = 7084			*N* = 6682^e^			*N* = 6039^e^			*N* = 5166^e^		
Characteristics												
Year of survey:												
2006	53/791	6.7%	0.003	78/738	10.6%	0.04	116/659	17.6%	0.001	149/535	27.9%	< 0.0001
2008	67/1211	5.5%		104/1144	9.1%		138/1039	13.3%		229/891	25.7%	
2010	76/1160	6.6%		117/1081	10.8%		145/962	15.1%		165/804	20.5%	
2012	88/1381	6.4%		133/1286	10.3%		153/1149	13.3%		181/982	18.4%	
2014	58/1321	4.4%		109/1259	8.7%		132/1145	11.5%		140/1003	14.0%	
2016	44/1220	3.6%		86/1174	7.3%		123/1085	11.3%		122/951	12.8%	
Sex:												
Female	188/3445	5.5%	0.98	316/3250	9.7%	0.35	404/2927	13.8%	0.34	449/2486	18.1%	0.07
Male	198/3639	7.5%		311/3432	9.1%		403/3112	12.9%		537/2680	20.0%	
Maternal age at child’s birth												
< 20 years	3/71	4.2%	0.03	6/68	8.8%	0.74	8/61	13.1%	0.49	7/52	13.5%	0.47
20–29 years	168/3107	5.4%		286/2929	9.8%		334/2635	12.7%		426/2266	18.8%	
30–39 years	192/3685	5.2%		318/3487	9.1%		434/3163	13.7%		520/2701	19.3%	
≥ 40 years	20/197	10.2%		14/177	7.9%		26/162	16.0%		31/135	23.0%	
Unknown / missing	3/24	12.5%		3 /21	14.3%		5/18	27.8%		2/13	15.4%	
Mother’s language^f^:												
French	250/4936	5.1%	0.03	400/4673	8.6%	0.004	525/4260	12.3%	0.001	655/3693	17.7%	0..04
English	32/389	8.2%		36/355	10.1%		32/317	10.1%		66/279	23.7%	
Other	47/942	5.0%		107/894	12.0%		131/787	16.6%		113/647	17.5%	
Unknown / missing	57/817	7.0%		84/760	11.1%		119/675	17.6%		152/547	27.8%	
Mother’s origin^f^:												
Canada	210/4066	5.2%	0.91	318/3845	8.3%	0.0004	415/3516	11.8%	0.0002	501/3063	16.4%	0.64
Other	52/990	5.3%		122/933	13.1%		134/809	16.6%		104/666	15.6%	
Unknown / missing	124/2028	6.1%		187/1904	9.8%		258/1714	15.1%		381/1437	26.5%	
Maternal level of education:												
< High school	40/490	8.2%	0.0008	55/449	12.2%	< 0.0001	77/390	19.7%	0.0013	63/303	20.8%	0.84
High school	101/1548	6.5%		175/1445	12.1%		172/1266	13.6%		209/1078	19.4%	
College	110/2074	5.3%		174/1958	8.9%		220/1780	12.4%		290/1533	18.9%	
University	129/2925	4.4%		218/2790	8.9%		332/2568	12.9%		417/2224	18.8%	
Unknown / missing	6/47	12.8%		5/40	12.5%		6/35	17.1%		7/28	25.0%	
Child rank:												
First	118/3118	3.8%	< 0.0001	206/2995	6.9%	< 0.0001	300/2784	10.8%	< 0.0001	437/2465	17.7%	0.01
≥ 2	260/3885	6.7%		405/3615	11.2%		501/3200	15.7%		542/2654	20.4%	
Unknown / missing	8/81	9.9%		16/72	22.2%		6/55	10.9%		7/47	14.3%	
Daycare attendance:												
Yes	251/5461	4.6%	< 0.0001	461/5201	8.9%	0.008	618/4732	13.1%	0.20	760/4066	18.7%	0.21
No	132/1583	8.3%		161/1444	11.1%		184/1275	14.4%		219/1075	20.4%	
Unknown / missing	3/40	7.5%		5/37	13.5%		5/32	15.6%		7/25	28.0%	
Child with a chronic disease:												
Yes	23/394	7.8%	0.69	46/369	12.5%	0.04	53/323	16.4%	0.09	65/265	24.5%	0.02
No	357/6650	5.4%		577/6279	9.2%		747/5686	13.1%		917/4878	18.8%	
Unknown / missing	6/40	15.0%		4/34	11.8%		7/30	23.3%		4/23	17.4%	
Gestational age at birth:												
< 37 weeks	35/511	6.8%	0.14	56/476	11.8%	0.04	60/419	14.3%	0.48	86/354	24.3%	0.009
≥ 37 weeks	336/6338	5.3%		534/5988	8.9%		713/5439	13.1%		870/4667	18.6%	
Unknown / missing	15/235	6.4%		37/218	17.0%		34/181	18.8%		30/145	20.7%	
Living with a partner												
Yes	339/6504	5.2%	0.004	559/6152	9.1%	0.01	731/5580	13.1%	0.28	901/4795	18.8%	0.11
No	40/480	8.3%		86/438	19.6%		57/379	15.0%		70/312	22.4%	
Unknown / missing	7/100	7.0%		12/92	13.0%		19/80	23.8%		15/59	25.4%	
Birth attendant^f^:												
Physician	221/4791	4.6%	< 0.0001	418/4557	9.2%	0.55	516/4126	12.5%	0.05	572/3567	16.0%	0.30
Midwife or other professionnal	38/252	15.1%		22/212	10.4%		33/189	17.5%		29/151	19.2%	
Unknown / missing	127/2041	6.2%		187/1914	9.8%		258/1724	15.0%		385/1448	26.6%	
Vaccine provider (at 2 months):												
Public health clinics	277/5033	5.5%	0.68	468/4742	9.9%	0.02	595/4264	14.0%	0.02	610/3629	16.8%	< 0.0001
Medical clinic/Hospital	105/1999	5.3%		153/1892	8.1%		204/1734	11.8%		367/1504	24.4%	
Unknown / missing	4/52	7.7%		6/48	12.5%		8/41	19.5%		9/33	27.3%	
Main vaccine provider (all vaccines):												
Public health clinics only										574/3473	16.5%	< 0.0001
Medical clinic/Hospital or both settings										406/1669	24.3%	
Unknown / missing										6/24	25.0%	
Missed opportunities at the 2-month visit:												
Yes	48/217	22.1%	< 0.0001	20/166	12.0%	< 0.0001	27/146	18.5%	0.07	27/114	23.7%	0.21
No	338/6867	4.9%		607/6516	9.3%		780/5893	13.2%		959/5052	19.0%	
Missed opportunities at the 12-month visit:												
Yes										227/702	32.3%	< 0.0001
No										759/4464	17.0%	

^a^ Analysis limited to children born in Quebec. Both cohorts included / ^b^ Correspond to the proportion of children who experienced new vaccine delays by each variable category / ^c^Chi-square test, unknown and missing excluded

^d^Correspond to the difference between 7183 and the number of children with a dose not administered

^e^For DTaP2 the 6682 represent 6698 children without vaccine delay for DTaP1 minus 16 children who had not received DTaP 2. For DTaP3 the 6039 represent 6055 children without vaccine delay for DTaP1 and DTaP2 minus 16 children who had not received DTaP 3. For MMR1 the 5166 represent 5232 children without vaccine delay for DTaP1, DTaP2 and DTaP3 minus 66 children who had not received MMR1

^f^These variables were not collected in 2006 and 2008

### Impact of new vaccine delays on vaccination status by 24 months of age

Among children with and without a vaccine delay for the DTaP1 at 2 months, 46.4 and 11.1% respectively had an incomplete vaccination status by 24 months for an unadjusted risk difference of 35.3% and a 27.7% adjusted risk difference (RD) (Table 3). The adjusted risk difference for incomplete vaccination status by 24 months was 13.8, 10.5 and 8.8% for new vaccine delays at 4, 6 and 12 months respectively. The relative risk of incomplete vaccination status by 24 months of age between children with and without a vaccine delay varied between 2.3 and 3.6 with overlapping confidence intervals. As computed with predicted probabilities from the multivariable models, 72.5% of 2-year children with an incomplete status by 24 months were attributable to a vaccine delay: 16.1% were attributable to a vaccine delay that first occurred at 2 months, 10.6% at 4 months, 14.0% at 6 months and 31.8% at 12 months. There was no risk-ratio heterogeneity for the survey year.

**Table 3:** Proportion of incomplete vaccination status by 24 months of age attributable to new vaccine delays, 2006–2016 (*N* = 3508)^a^

Vaccination visits	Incomplete vaccination status		Risk difference for incomplete vaccination status		Relative risk of incomplete vaccination status	% of cases exposed	Attributable risk in the population
	With a new vaccine delay % (n/N)	Without a vaccine delay % (n/N)	Unadjusted	Adjusted^b^ (95% CI)	Adjusted^b^ (95% CI)		Adjusted (95% CI)^c^
DTaP at 2 months	46.4% (96/207)	11.1% (361/3250)	35.3%	27.7% (22.2%; 28.2%)	3.6 (2.95; 4.37)	21.0%	16.1% (8.4%; 23.7%)
DTaP at 4 months	24.3% (64/263)	9.8% (292/2982)	14.5%	13.8% (13.4%; 14.0%)	2.5 (1.91; 3.18)	18.0%	10.6% (1.4%; 19.3%)
DTaP at 6 months	19.6% (71/362)	8.3% (217/2616)	11.3%	10.5% (10.3%; 10.8%)	2.3 (1.81; 3.02)	24.7%	14% (3.9%; 23.5%)
Measles at 12 months	17.5% (99/567)	4.6% (94/2025)	12.8%	8.8% (8.5%; 9.0%)	2.9 (2.12; 3.85)	51.3%	31.8% (20.5%; 40.1%)

*CI* Confidence interval estimated using bootstrap with 1000 replications

^a^Analysis restricted to the 2-year cohort and to children born in Quebec. Unvaccinated children: 51 at 2 months, 5 at 4 months, 4 at 6 months and 24 at 12 months

^b^Risk difference estimated with standardization using predicted probabilities calculated from Robust Poisson multivariable regression. Incomplete vaccination for common antigens only (ie. DTaP-VPI-Hib. MMR and Men-C-C vaccines). Risk difference and relative risk adjusted for survey year, mother’s age at child birth, sex, mother’s education, child’s rank, daycare attendance, child’s health condition, gestational age, family type, main vaccine provider, missed opportunities. Analysis restricted to children with no vaccine delays at previous dose. Mother’s language, mother’s country of origin and the birth attendant were excluded from this analysis as they were not collected in 2006 and 2008 (more than 10% of missing)

^c^Similar results were observed using the following formula: $\mathrm{ARp}=\frac{\mathrm{PCE}\ast \left(\mathrm{RR}-1\right)}{\mathrm{RR}}$ PCE = proportion of cases exposed (i.e. proportion of children with incomplete vaccination status with new vaccine delays)

### Factors associated with new vaccine delays

Compared to the firstborn, other children were more likely to have a new vaccine delay at 2, 4, 6 and 12 months (Table 4). Missed opportunity at the 2-month visit was the most important factor associated with vaccine delays for DTaP1 (RR 4.28 (IC 3.18; 5.77)). At 2 months, children who were not attending daycare (versus attending daycare) and children whose parent who completed the survey was living without a partner (versus with a partner) were more likely to experience delays. Vaccination in medical clinic/hospital (versus in public health clinics) at the 2-month visit was associated with lower risk of vaccine delay for DTaP2 and DTaP3. The risk of vaccine delays for DTaP1 and DTaP2 decreased with higher maternal education. Missed opportunity at the 12-month visit was the most important factor associated with vaccine delays for measles at 12 months. Gestational age at birth lower than 37 weeks (vs ≥ 37 weeks) was also associated with vaccine delays at 12 months. Finally, in contrast to results observed for DTaP2 and DTaP3, children vaccinated in medical clinic/hospital or in both settings for the 12-month visit were more likely to experience measles vaccine delays at 12 months compared with children vaccinated in public health clinics.

**Table 4:** Factors significantly associated with new vaccine delays at 2–4-6 and 12 months of age, 2006–2016 (*N* = 7183)^a^

Factors associated with vaccine delays^b^	DTaP1 at 2 months		DTaP2 at 4 months^c^		DTaP3 at 6 months^d^		Measles at 12 months^e^	
	Unadjusted RR (95% CI)	Adjusted RR (95% CI)	Unadjusted RR (95% CI)	Adjusted RR (95% CI)	Unadjusted RR (95% CI)	Adjusted RR (95% CI)	Unadjusted RR (95% CI)	Adjusted RR (95% CI)
Year of survey:								
2006	1.86 (1.26; 2.74)	1.38 (0.91; 2.09)	1.44 (1.08;1.93)	1.56 (1.15; 2.12)	1.55 (1.23; 1.96)	1.71 (1.35; 2.17)	2.17 (1.75; 2.69)	1.84 (1.45; 2.32)
2008	1.53 (1.06; 2.23)	1.20 (0.82; 1.75)	1.24 (0.94; 1.63)	1.29 (0.97; 1.71)	1.17 (0.93; 1.47)	1.24 (0.98; 1.56)	2.00 (1.64; 2.45)	1.74 (1.41; 2.15)
2010	1.82 (1.26; 2.61)	1.55 (1.08; 2.22)	1.48 (1.13; 1.93)	1.53 (1.17; 2.02)	1.33 (1.06; 1.66)	1.40 (1.12; 1.76)	1.60 (1.29; 1.98)	1.53 (1.23; 1.90)
2012	1.77 (1.24; 2.52)	1.57 (1.11; 2.24)	1.41 (1.09; 1.83)	1.38 (1.06; 1.81)	1.18 (0.94; 1.47)	1.20 (0.96; 1.51)	1.44 (1.16; 1.78)	1.39 (1.12; 1.73)
2014	1.22 (0.83;1.79)	1.13 (0.78; 1.66)	1.18 (0.90; 1.55)	1.20 (0.91; 1.58)	1.02 (0.81; 1.28)	1.02 (0.81; 1.29)	1.09 (0.87; 1.36)	1.09 (0.87; 1.37)
2016 *(reference)*	1	1	1	1	1	1	1	1
Maternal level of education for each increase of one level	0.82 (0.74; 0.90)	0.90 (0.81; 0.99)	0.83 (0.77; 0.90)	0.86 (0.79; 0.92)				
Child’s rank:								
≥ 2	1.72 (1.39; 2.12)	1.68 (1.36; 2.09)	1.63 (1.39; 1.91)	1.63 (1.39; 1.92)	1.45 (1.27; 1.66)	1.47 (1.28; 1.68)	1.15 (1.03; 1.30)	1.16 (1.03; 1.30)
First *(reference)*	1	1		1		1		1
Gestational age at birth								
< 37 weeks							1.30 (1.58; 1.07)	1.26 (1.04; 1.53)
≥ 37 weeks *(reference)*							1	1
Daycare attendance:								
No	1.81 (1.48; 2.22)	1.48 (1.20; 1.84)						
Yes *(reference)*	1	1						
Living with a partner:								
No	1.60 (1.17; 2.19)	1.48 (1.07; 2.05)						
Yes *(reference)*	1	1						
Vaccine provider (2-months):								
Medical clinic or Hospital			0.82 (0.69; 0.98)	0.80 (0.67; 0.96)	0.84 (0.73; 0.98)	0.79 (0.68; 0.92)		
Public health clinics *(reference)*			1	1	1	1		
Main vaccine provider (all vaccines):								
Medical clinic/ Hospital or both settings							1.47 (1.32; 1.65)	1.22 (1.08; 1.39)
Public health clinics only *(reference)*							1	1
Missed opportunities at the 2-month visit:								
Yes	4.49 (3.43; 5.89)	4.28 (3.18; 5.77)						
No *(reference)*	1	1						
Missed opportunities at the 12-month visit:								
Yes							1.90 (1.68; 2.16)	1.47 (1.26; 1.71)
No *(reference)*							1	1

*RR* relative risk, *CI* confidence interval

^a^ Both cohorts were included in this analysis. Analysis limited to children born in Quebec. Multivariable analysis exclude children unvaccinated since birth (*n* = 43 for 1-year cohort and *n* = 42 for 2-year cohort)

^b^Factors selected with robust Poisson multivariable regression with backward selection. Mother’s language, mother’s country of origin and the birth attendant were excluded from this analysis as they were not collected in 2006 and 2008 (more than 10% of missing). For each visit, results are presented only for covariates significant in the models

^c^Restricted to children without vaccine delay at 2 month vaccines /^d^ Restricted to children without vaccine delay at 2 and 4 month vaccines / ^e^Restricted to children without vaccine delay at 2, 4 and 6 month vaccines

## Discussion

While the literature on vaccine coverage put an emphasis on vaccine delays at 2 months, this study shows that the proportion of vaccine delays was greater at later visits increasing from 5.4% at 2 months, to 13.3% at 4 months, to 23.1% at 6 months and to 23.6% at 12 months. Vaccine delay at one visit had an impact on the timing and administration of subsequent doses as most children with vaccine delay for a DTaP dose received their subsequent dose with a 2-month interval rather than the shortened interval recommended in the accelerated schedule. A vaccine delay decreased the probability of having received all recommended vaccines by 24 months of age and 72.5% of incomplete status by 24 months of age were attributed to a new vaccine delay at 2, 4, 6 or 12 months (16.1% at 2 months, 10.6% at 4 months, 14.0% at 6 months and 31.8% at 12 months).

This study found that delays at the first visit did contribute to incomplete vaccination status by 24 months of age and that more than 75% of children with a vaccine delay at 2-months also had vaccine delays at later visits including a third with vaccine delays at all next three visits. Association between vaccine delays at the first vaccination visit and later vaccine delays or incomplete vaccination status by 24 months of age has been widely reported [9, 16–18]. While other studies also identified that vaccine delays increased at subsequent vaccine visits [9, 11, 34, 35], they did not assess their impact on incomplete vaccination status by 24 months, the additional step this study provides.

The interventions deployed in public health clinics in Quebec since 2006 to improve timeliness at the 2 and 12 month visits seem to have successfully reduced vaccine delays at all visits. While only 17% of 2-year-old children had received all recommended doses with no delay in 2006, this proportion had increased to 50% in 2016 [15, 23]. In a cohort of 361,901 children born from 2004 through 2012 in United States, 55% of children had been fully vaccinated with no delay by 24 months of age [36]. Reminders or recall interventions sent by vaccine providers are effective to increase the likelihood of being vaccinated and to reduce the number of underimmunized days [37, 38]. In the current study, for children vaccinated in public health clinics, the proportion with new vaccine delays decreased from 2006 to 2016 for each vaccination visits: a decrease of 5.5% at 2 months, 7.2% at 4 months, 9.3% at 6 months and 19.8% at 12 months. The risk of vaccine delays for the 12 month visit was 1.22 times higher for children vaccinated in medical clinic/hospital versus those vaccinated in public health clinics. In contrast, in medical clinic/hospital where no intervention was done, no major changes have been observed in the proportion of new vaccine delays from 2006 to 2016. In public health clinics, efforts to reduced vaccine delays were more important for the 2 and 12 month visits and the proportions of children with vaccine delays at 4 and 6 months remained higher in this setting compared with medical clinic/hospital for each survey year except in 2016, resulting in a higher risk of vaccine delays.

In the present study, children who are not the firstborn and those with missed opportunities at the 2-month and the 12-month visits were more likely to had vaccine delays. These two factors have been frequently associated with vaccine delays in other similar studies [6, 10, 34, 39]. A high number of children in the household may impact the accessibility to healthcare settings, including vaccination services. In addition, children with older siblings are possibly more exposed to minor illness, resulting in missed opportunities and vaccine delays [40–42]. We observed that children of single parent (versus living with a partner) had a greater risk for vaccine delays at 2-months and these results might be associated with constraints to access vaccination services [6, 34]. In the current study, the risk of vaccine delays for DTaP1 and DTaP2 decreases with higher maternal education. The literature is inconsistent on this issue; some authors found an association similar to ours while others observed a higher risk of delays with an increasing education level [6, 10]. A literature review found that higher maternal level of education was associated with vaccine hesitancy which is itself associated with vaccine delays [43]. This subject may require future studies.

This study has some limitations. The response rate to vaccine coverage surveys varied from 61 to 73% depending upon the year and cohort. Participants may have had more positive behaviours regarding vaccination than non-participants, resulting in less vaccine delays and higher vaccine coverage. Validation of the information on immunization from questionnaires was restricted to children whose information was not consistent with the provincial vaccine schedule. We cannot rule out that this practice has led to an overestimation of vaccine coverage, but the impact on our results might be minimal as vaccination card compared to medical chart usually has a good positive predictive value [44, 45]. As only written data were accepted in this study, underestimation of vaccine coverage cannot be ruled out but is unlikely as many sources were consulted to obtain vaccine information. As markers of timeliness, vaccine delays were estimated only for DTaP and measles containing vaccines despite other vaccines being administered at 2, 4, 6 and 12 months. While most recommended vaccines are administered at the same visit, the burden associated with vaccine delays presented in this analysis has probably been underestimated.

The current study estimated the risk of incomplete vaccination status by 24 months attributable to vaccine delays at different ages an information that can be useful to decide or prioritize public health interventions. However, the interpretation of ARp as the fraction of the outcome that could be eliminated if exposure could be totally removed from the population is valid only under certain conditions [46]. First, exposure has to be causal rather than merely associated with the disease. By estimating counterfactual risk with model-based standardization, we attempted to estimate the average causal effect of new vaccine delays at 2, 4, 6 or 12 months on vaccination status by 24 months. However, root causes of vaccine delays remained unknown and the elimination of vaccine delays may not result in an improvement of complete vaccination status by 24 months as large as estimated by ARp. Second, estimation of ARp has to be unbiased. While multivariable models included most of the determinants identified in the literature, as for any observational study, residual confounding cannot be ruled out. Finally, the elimination of exposure has to be without any effect on distribution of other risk factors. As other factors included in this analysis are mainly unmodifiable risk factors, it is unlikely that the elimination of vaccine delays has any effect on their distribution. As the attributable risk depends upon the prevalence of vaccine delays at various visits, which may vary between populations, this limits the generalizability of this result to other jurisdictions [46].

## Conclusion

While great emphasis has been put on vaccine delays at the first vaccination visit, the prevalence of vaccine delays in this study was greater with later visits and most of incomplete vaccination status by 24 months of age was associated with vaccine delays occurring after the 2 month visit. Interventions to improve timeliness deployed in public health clinics in Quebec seem to have reduced vaccine delays at all visits from 2006 to 2016. These interventions should also focus on other vaccine visits which contributed to the burden associated with vaccine delays and on more vulnerable groups identified in this study. Accelerated vaccination schedules recommended for children with vaccine delays may prevent further delays and reduce their impact.

## Additional files

Additional file 1: Characteristics of children participants, 1-year and 2-year cohorts, 2006–2016 (*n* = 7183). (DOCX 20 kb)

Additional file 2: Intervals following the next vaccine dose for children with new vaccine delays at the DTaP1 and DTaP2. (DOCX 26 kb)

Additional file 3: New vaccine delays at 2, 4, 6 and 12 months by vaccine provider and survey year, 1-year and 2-year cohorts, 2006–2016. (DOCX 17 kb)

## Abbreviations

ARp: Attributable risk in the population
CI: Confidence interval
DTaP: Diphtheria, tetanus, acellular pertussis and polio containing vaccine
DTaP-IPV-Hib: Diphtheria, tetanus, acellular pertussis, polio virus and *Haemophilus Influenzae* type b vaccine
DTaP-IPV-Hib-HB: Diphtheria, tetanus, acellular pertussis, polio virus, *Haemophilus Influenzae* type b vaccine and Hepatitis B vaccine
Measles: Measles-containing vaccine
Men-C-C: Meningococcal C conjugate vaccine
MMR: Measles, mumps and rubella vaccine
MMRV: Measles, mumps, rubella and varicella vaccine
PCV: Pneumococcal conjugate vaccine
Rota: Rotavirus vaccine
RR: Risk ratio
VPD: Vaccine-preventable diseases
