# Supplementary material for: Impact of vaccine delays at the 2, 4, 6 and 12 month visits on incomplete vaccination status by 24 months of age in Quebec, Canada
Source: BMC Public Health. 2018 Dec 11;18:1364. doi: 10.1186/s12889-018-6235-6 (PMC6288945; doi:10.1186/s12889-018-6235-6)
Supplement: Supplementary file 1 — Characteristics of children participants, 1-year and 2-year cohorts, 2006–2016 (n = 7183). (DOCX 20 kb) [file 12889_2018_6235_MOESM1_ESM.docx]

| **Characteristics of children participants, 1-year and 2-year cohorts, 2006-2016 (n=7183)^§^** | | |
| --- | --- | --- |
|  | n | % |
| **Characteristics** |  |  |
| **Year of survey** |  |  |
| 2006 | 791 | 11.0 |
| 2008 | 1231 | 17.1 |
| 2010 | 1172 | 16.3 |
| 2012 | 1418 | 19.7 |
| 2014 | 1334 | 18.6 |
| 2016 | 1237 | 17.2 |
| **Cohort** |  |  |
| 1-year cohort | 3675 | 51.2 |
| 2-year cochort | 3508 | 48.8 |
| **Sex** |  |  |
| Female | 3497 | 48.7 |
| Male | 3686 | 51.3 |
| **Mother's age at child birth** |  |  |
| < 20 years | 72 | 1.0 |
| 20-29 years | 3157 | 44.0 |
| 30-39 years | 3758 | 51.9 |
| > 40 years | 202 | 2.8 |
| unknown/missing | 24 | 0.3 |
| **Mother's language*** |  |  |
| French | 5027 | 70.0 |
| English | 393 | 5.5 |
| Others | 946 | 13.3 |
| unknown/missing | 817 | 11.4 |
| **Mother's origin*** |  |  |
| Canada | 4138 | 57.6 |
| Others | 997 | 13.9 |
| unknown/missing | 2048 | 28.5 |
| **Mother's level of education** |  |  |
| < secondary school | 497 | 6.9 |
| Completed Secondary school | 1577 | 22.0 |
| Completed College school | 2106 | 29.3 |
| Completed University school | 2956 | 41.2 |
| unknown/missing | 47 | 0.7 |
| **Child's rank** |  |  |
| First | 3146 | 43.8 |
| ≥ 2 | 3956 | 55.1 |
| unknown/missing | 81 | 1.1 |
| **Daycare attendance** |  |  |
| Yes | 5525 | 76.9 |
| No | 1618 | 22.5 |
| unknown/missing | 40 | 0.6 |
| **Child with a health condition** |  |  |
| No | 6733 | 93.7 |
| Yes | 400 | 5.6 |
| unknown/missing | 50 | 0.7 |
| **Gestational age at birth** |  |  |
| < 37 weeks | 513 | 7.1 |
| ≥ 37 weeks | 6424 | 89.4 |
| unknown/missing | 246 | 3.4 |
| **Living with a partner** |  |  |
| Yes | 6598 | 91.9 |
| No | 484 | 6.7 |
| unknown/missing | 101 | 1.4 |
| **Birth attendant*** |  |  |
| Doctor | 4831 | 67.3 |
| Midwife/Others | 291 | 4.1 |
| unknown/missing | 2061 | 28.7 |
| **Main vaccine provider** |  |  |
| Public health clinics only | 4805 | 66.7 |
| Hospital/medical clinic only or both | 2246 | 31.3 |
| unknown/missing | 132 | 1,8 |
| **Missed opportunities at the 2-month visit** |  |  |
| No | 6879 | 95.8 |
| Yes | 219 | 3.1 |
| Unvaccinated | 85 | 1.2 |
| **Missed opportunities at the 12-month visit** |  |  |
| No | 6033 | 84.0 |
| Yes | 1065 | 14.8 |
| Unvaccinated | 85 | 1.2 |
| **Delayed 2-month vaccines (delay ≥ 30 days)** |  |  |
| No | 6698 | 93.2 |
| Yes | 386 | 5.4 |
| Unvaccinated or first DTaP unreceived | 99 | 1.4 |
| **Delayed 4-month vaccines (delay ≥ 30 days)** |  |  |
| No | 6112 | 85,1 |
| Yes | 941 | 13.1 |
| Unvaccinated or second DTaP unreceived | 130 | 1,8 |
| **Delayed 6-month vaccines (delay ≥ 30 days)** |  |  |
| No | 5385 | 75,0 |
| Yes | 1617 | 22.5 |
| Unvaccinated or third DTaP unreceived | 181 | 2.5 |
| **Delayed 12-month vaccines (delay ≥ 30 days)** |  |  |
| No | 5277 | 74,5 |
| Yes | 1634 | 22.7 |
| Unvaccinated or first measles-containing vaccine unreceived | 272 | 3,8 |

^§^ Analysis limited to children born in Quebec.

* These variables were not collected in 2006 and 2008.
