# Supplementary material for: Impact of vaccine delays at the 2, 4, 6 and 12 month visits on incomplete vaccination status by 24 months of age in Quebec, Canada
Source: BMC Public Health. 2018 Dec 11;18:1364. doi: 10.1186/s12889-018-6235-6 (PMC6288945; doi:10.1186/s12889-018-6235-6)
Supplement: Supplementary file 2 — Intervals following the next vaccine dose for children with new vaccine delays at the DTaP1 and DTaP2. (DOCX 26 kb) [file 12889_2018_6235_MOESM2_ESM.docx]

**Intervals between the administration of DTaP1 (2 months) and DTaP2 (4 months) among children with vaccine delays at the DTaP1 who received the DTaP2 (n=371)**

57%

20%

**Intervals between the administration of DTaP2 (4 months) and DTaP3 (6 months), among children with new vaccine delays at the DTaP2 who received the DTaP3 (n=614)**
