# Supplementary material for: Impact of vaccine delays at the 2, 4, 6 and 12 month visits on incomplete vaccination status by 24 months of age in Quebec, Canada
Source: BMC Public Health. 2018 Dec 11;18:1364. doi: 10.1186/s12889-018-6235-6 (PMC6288945; doi:10.1186/s12889-018-6235-6)
Supplement: Supplementary file 3 — New vaccine delays at 2, 4, 6 and 12 months by vaccine provider and survey year, 1-year and 2-year cohorts, 2006–2016. (DOCX 17 kb) [file 12889_2018_6235_MOESM3_ESM.docx]

**New vaccine delays at 2, 4, 6 and 12 months by vaccine provider and survey year, 1-year and 2-year cohorts, 2006-2016^§^**

|  |  | Public health clinic only | | | Medical clinic/hospital or both settings | | |
| --- | --- | --- | --- | --- | --- | --- | --- |
|  | Survey year | Frequency | % | P value* | Frequency | % | P value* |
| **Vaccine delays DTaP1** |  |  |  |  |  |  |  |
| **(2 months)** | 2006 | 42/464 | 9.1% | < 0.0001 | 10/311 | 3.2% | 0.002 |
| Unknown/missing n=52** | 2008 | 56/762 | 7.4% |  | 11/439 | 2.5% |  |
|  | 2010 | 48/804 | 6.0% |  | 27/349 | 7.7% |  |
|  | 2012 | 58/994 | 5.8% |  | 29/385 | 7.5% |  |
|  | 2014 | 38/1029 | 3.7% |  | 19/284 | 6.7% |  |
|  | 2016 | 35/980 | 3.6% |  | 9/231 | 3.9% |  |
| **New vaccine delays DTaP2** |  |  |  |  |  |  |  |
| **(4 months)** | 2006 | 58/422 | 13.7% | 0.0001 | 19/301 | 6.3% | 0.69 |
| Unknown/missing n=48** | 2008 | 66/706 | 9.4% |  | 37/428 | 8.6% |  |
|  | 2010 | 90/753 | 12.0% |  | 27/322 | 8.4% |  |
|  | 2012 | 102/929 | 11% |  | 30/356 | 8.4% |  |
|  | 2014 | 91/989 | 9.2% |  | 18/263 | 6.8% |  |
|  | 2016 | 61/943 | 6.5% |  | 22/222 | 9.9% |  |
| **New vaccine delays DTaP3** |  |  |  |  |  |  |  |
| **(6 months)** | 2006 | 74/364 | 20.3% | 0.0002 | 41/281 | 14.6% | 0.62 |
| Unknown/missing n=41** | 2008 | 91/640 | 14.2% |  | 46/390 | 11.8% |  |
|  | 2010 | 109/662 | 16.5% |  | 34/295 | 11.5% |  |
|  | 2012 | 116/825 | 14.1% |  | 37/324 | 11.4% |  |
|  | 2014 | 108/894 | 12.1% |  | 23/244 | 9.4% |  |
|  | 2016 | 97/879 | 11.0% |  | 23/200 | 11.5% |  |
| **New vaccine delays measles** |  |  |  |  |  |  |  |
| **(12 months)** | 2006 | 85/274 | 31.0% | <0.0001 | 61/252 | 24.2% | 0.008 |
| Unknown/missing n=24** | 2008 | 123/516 | 23.8% |  | 105/368 | 28.5% |  |
|  | 2010 | 78/514 | 15.2% |  | 86/287 | 30.0% |  |
|  | 2012 | 116/661 | 17.6% |  | 65/321 | 20.3% |  |
|  | 2014 | 87/748 | 11.6% |  | 52/252 | 20.6% |  |
|  | 2016 | 85/760 | 11.2% |  | 37/189 | 19.6% |  |

**^§^** Analysis limited to children born in Quebec (Canada). Both cohorts included. For 99 children, DTaP1 was not administered, 130 for DTaP2, 181 for DTaP3 and 272 for MMR1. *Chi-square test. ** Vaccine providers unknown/missing.
